# CAISMOV24, a new human low-grade serous ovarian carcinoma cell line

**DOI:** 10.1186/s12885-017-3716-4

**Published:** 2017-11-13

**Authors:** Rodrigo Fernandes da Silva, Daniela Maira Cardozo, Gisele Olinto Libanio Rodrigues, Caroline Natânia de Souza-Araújo, Natacha Azussa Migita, Liliana Aparecida Lucci de Angelo Andrade, Sophie Derchain, José Andrés Yunes, Fernando Guimarães

**Affiliations:** 1 Faculdade de Ciências Médicas, University of Campinas, Campinas, SP Brazil; 2 Instituto de Biologia, University of Campinas, Campinas, SP Brazil; 3 Laboratório de Biologia Molecular, Centro Infantil Boldrini, Campinas, SP Brazil; 4 Women’s Hospital “Professor Doutor José Aristodemo Pinotti” – CAISM, University of Campinas, Rua Alexander Fleming 101, Campinas, SP 13083-881 Brazil

**Keywords:** Ascites, Cell culture, Comparative genomic hybridization, KRAS

## Abstract

**Background:**

The spontaneous immortalization of primary malignant cells is frequently assigned to their genetic instability during in vitro culturing. In this study, the new epithelial ovarian cancer cell line CAISMOV24 was described and compared with its original low-grade serous ovarian carcinoma.

**Methods:**

The in vitro culture was established with cells isolated from ascites of a 60-year-old female patient with recurrent ovarian cancer. The CAISMOV24 line was assessed for cell growth, production of soluble biomarkers, expression of surface molecules and screened for typical mutations found in serous ovarian carcinoma. Additionally, comparative genomic hybridization was employed to compare genomic alterations between the CAISMOV24 cell line and its primary malignant cells.

**Results:**

CAISMOV24 has been in continuous culture for more than 30 months and more than 100 in vitro passages. The cell surface molecules EpCAM, PVR and CD73 are overexpressed on CAISMOV24 cells compared to the primary malignant cells. CAISMOV24 continues to produce CA125 and HE4 in vitro. Although the cell line had developed alongside the accumulation of genomic alterations (28 CNV in primary cells and 37 CNV in CAISMOV24), most of them were related to CNVs already present in primary malignant cells. CAISMOV24 cell line harbored *KRAS* mutation with wild type *TP53*, therefore it is characterized as low-grade serous carcinoma.

**Conclusion:**

Our results corroborate with the idea that genomic alterations, depicted by CNVs, can be used for subtyping epithelial ovarian carcinomas. Additionally, CAISMOV24 cell line was characterized as a low-grade serous ovarian carcinoma, which still resembles its primary malignant cells.

**Electronic supplementary material:**

The online version of this article (10.1186/s12885-017-3716-4) contains supplementary material, which is available to authorized users.

## Background

Ovarian cancer is the most lethal gynecological cancer, causing most of women’s death. Its lethality is a consequence of the lack of symptoms or biomarkers enabling the early diagnosis of disease, and the propensity of malignant cells to seed the peritoneal cavity. The epithelium accounts for 90% of ovarian cancers [1, 2]. Epithelial ovarian cancer (EOC) comprises four major histotypes, which are further classified based on their growth and molecular characteristics in slowly developing or type I tumors, and more aggressive or type II tumors [3–5]. The serous histotype is responsible for almost 70% of epithelial ovarian cancers, which are additionally differentiated into low (type I) and high grades (type II). The most aggressive subtype of serous ovarian carcinoma accounts for two-thirds of all ovarian cancer deaths, making them the most extensively studied ovarian carcinoma [3, 4, 6].

Biomarkers and molecular characterization of the tumors can represent a more accurate biological classification to separate and treat these tumors, rather than their site of origin. As an example, cancer antigen 125 (CA125) and human epididymis protein 4 (HE4) have been detected in the serum of ovarian cancer patients. Together, they enhance the sensitivity and specificity for the diagnosis of the disease [7, 8]. Additionally, the identification of molecular surface markers on malignant cells can both contribute to the diagnosis and a better comprehension of tumor-host interactions [9–11]. Recently, molecular characteristics of tumors have become the new standard classification for clinical pathology. In this concern, methods such as comparative genomic hybridization allow the detection of deletions and duplications of genomic segments, known as copy number variation (CNV). This method has been used to evaluate differences between cancer histotypes and putative target driver genes in EOC [12, 13].

Cell lines from various tumor types have been developed for use as a tumor model for controlled laboratory studies. Usually, cell lines have the facility to be cultivated, stored and shared between different laboratories. However, cell lines are known for their susceptibility to genetic and metabolic alterations, which can alter their characteristics in relation to the primary malignant cells. Ideally, the more a cell line resembles the primary malignant cells, the more useful it will be for scientific research. In vitro cultures of primary malignant cells and established cell lines have been widely employed as experimental models for the understanding of ovarian cancer biology, the evaluation of new therapeutic approaches and to search for tumor markers [14–17]. Currently, Broad-Novartis Cancer Cell Line Encyclopedia (CCLE https://portals.broadinstitute.org/ccle) includes 52 cell lines of human ovarian cancer [18]. Jacob et al. [17], recently published a review including 104 human cell lines originating from ovarian cancers or human ovarian surface epithelium. However, there are a limited number of the already established ovarian carcinoma cell lines, which have been well characterized as in vitro models. Domcke et al. [19] evaluated the genomic profiles of 47 ovarian cell lines in comparison to ovarian tumor samples, and found that some of the cell lines most commonly used as experimental models do not resemble their cognate tumor profile. Thus, new, well-characterized cell lines that resemble the different histological and molecular subtypes of ovarian neoplasia are still needed, particularly for low-grade serous ovarian carcinoma [3, 4]. The goal of this study was to report the establishment of a new human epithelial ovarian carcinoma cell line (CAISMOV24), describing its phenotypic and molecular characteristics, and comparing genomic alterations between the cell line and its primary malignant cells.

## Methods

### Patient and ascites sampling

**Fig. 1:**
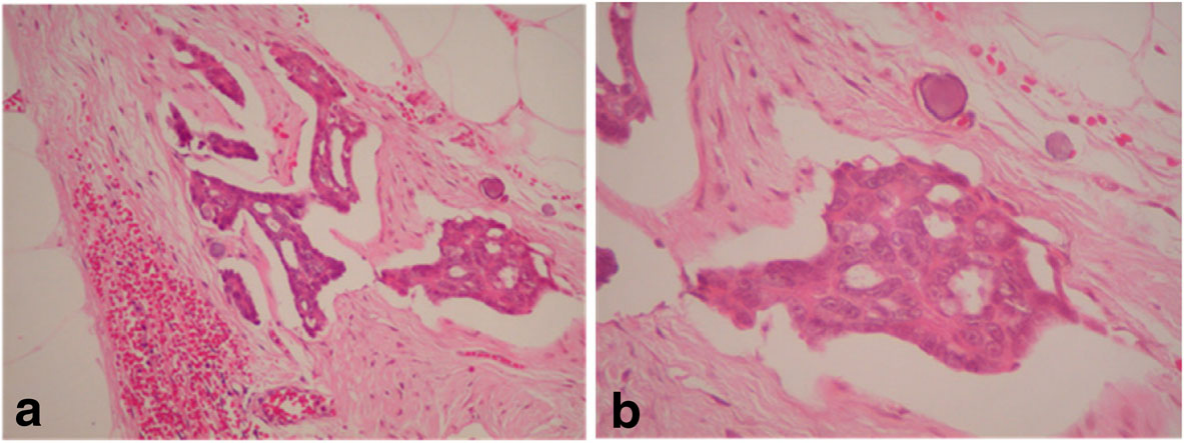
Peritoneal implant of low-grade serous ovarian carcinoma. **a** obj 10× and **b** obj 40×

The patient was a 60-year-old woman, originally subjected to exploratory laparotomy for the diagnosis of ovarian neoplasia, procedure that was carried out at "Hospital da Mulher Prof. Dr. José Aristodemo Pinotti–Centro de Atenção Integral à Saúde da Mulher", the Women’s Hospital of the University of Campinas (Campinas, Brazil). A non-resectable stage IIIC ovarian neoplasia was found from which a biopsy of the tumor mass was collected. The anatomical pathological diagnosis was consistent with a low-grade serous adenocarcinoma of the ovary (Fig. 1). Subsequently, the patient was treated with chemotherapy followed by surgery for cytoreduction.

Relapse occurred 1 year later, when ascites fluid originally used to initiate the in vitro culture of EOC cells was collected by sterile aspiration at the time of a laparotomy, in May 2011. The serum level of CA125 at the time of ascites collection was 2216 U/ml. The patient died in August 2012 with pulmonary metastasis. The study was approved by the Research Ethics Committee of Unicamp (897/2011) and was registered on the Brazilian National Health Council (CAAE: 0807.0.146.000–11). Signed informed consent was obtained from the patient prior sampling of ascites and blood.

### Initiation of the cell culture and maintenance of the cell line

The ascites fluid was taken to our cell culture laboratory and processed immediately after sampling. Ascites fluid was transferred to 50 ml conical tubes, the total volume was doubled by the addition of balanced salt solution (PBS; Nutricell, Campinas, Brazil) and 25 IU/ml of heparin was added (Liquemine, Roche, São Paulo, SP, Brazil). The cell fraction was isolated by centrifugation and washed twice in PBS (400 g/8 min/room temperature). The final cell pellet was suspended in HAM F10 medium supplemented with L-glutamine (2 mM, Nutricell, Campinas, Brazil) and fetal bovine serum (10%, FBS; Nutricell, Campinas, Brazil). Cell number and viability were assessed using a hemocytometer and the trypan-blue exclusion method, respectively. Although the cell suspension was constituted by a diversity of cells at this time, the presence of tumor cells was noticed and the cell suspension was adjusted to 1 × 10^6^ cells/ml with culture medium. Thus, 10 × 10^4^ cells were seeded in petri dishes (35 X 10 mm; Corning, New York, USA), with supplemented culture medium added up to a volume of 2 ml (resulting in a 5 × 10^4^ cells/ml suspension); dishes were placed in an incubator (37 °C, 5% CO_2_). The initial subcultures (the first 12 in vitro passages) were performed without a regular time period, ranging from 3 to 4 weeks, after which cells were detached with trypsin/EDTA (0.25%; Nutricell, Campinas, Brazil), counted and seeded in new T25 flasks (JetBiofil, Guangzhou, China) at 10^4^ cells/cm^2^ with supplemented HAM F10 medium. From the 12th in vitro passage onwards, the maintenance of the cultures and subculturing became regular. Thus, cultures were replenished with fresh medium every 2–3 days, and treated with trypsin/EDTA for cell re-plating every 2 weeks.

### Assessment of in vitro growth kinetics of the cells

Quantification of the dividing capabilities of the cells was assessed several times during the 63rd and 100th in vitro passages. Thus, T25 culture flasks were seeded with 10^4^ cells/cm^2^. Subsequently, replicates of the culture flasks were treated with trypsin/EDTA for cell detaching at different time points, and their number and viability were assessed using a hemocytometer and the trypan-blue exclusion method. The mean number of cells per centimeter square was expressed on a semi-logarithmic scale. Additionally, cell division in cultures was assessed by flow cytometry. For this, CAISMOV24 cells were labelled with violet proliferation dye 450 (VPD450, Becton, Dickinson and Company, Mountain View, USA) according to the manufacturer’s instructions, prior to being seeded in T25 culture flasks at a density of 10^4^ cells/cm^2^. Subsequently, cells were detached at different time points (days 3, 5, 7 and 10) for acquisition in a FACS Verse with FACS Suite software (Becton Dickinson, San Jose, USA). Stained cell suspensions were analyzed by setting the appropriate SSC/FSC gate on tumor cells and taking into account the fluorescence intensities on day 0 in labelled and unlabeled cells. The proliferation platform of FlowJo 7.1 software (Tree Star, Ashland, USA) was used for the data analysis.

### Immunophenotyping of cell line and biomarkers production in culture

A flow cytometric-based assay was used according to standard procedures. Briefly, the cells were mixed with appropriate concentrations of fluorochrome-conjugated monoclonal antibodies (MAb), anti-CD326 (anti-EpCAM, clone EBA), anti-HLA-class I (clone G46–2.6), anti-TGFβ1 (clone TW4-9E7), anti-CD39 (clone TU66), anti-CD73 (clone A2D) (Becton, Dickinson and Company, Mountain View, USA) and anti-CD155 (anti-PVR, clone 2H7) (eBioscience, San Diego, USA). Cells were incubated for 30 min in an ice bath and protected from light. After incubation, cells were washed twice with PBS and the final cell pellets were suspended for acquisition in a FACS Verse with FACS Suite software (Becton Dickinson, San Jose, USA). The human erythromyeloblastoid cell line K562 was used as a negative control for most of the surface molecules assessed in the assay. All samples were analyzed by setting the appropriate SSC/FSC gate on the tumor cells. FlowJo 10.0 software (Tree Star, Ashland, USA) was used for the data analysis.

The levels of soluble tumor markers CA125 and HE4 (human epididymis protein 4) were evaluated in the culture media taken between the 45th and 65th in vitro passages of the cells. An automatic chemiluminescence-based immunoassay was employed for this purpose. Thus, the CA125 II and Architect HE4 kits, combined with the Architect iSystem (Abott Diagnostics; Wiesbaden, Germany), were used according to the manufacturer’s instructions to assess CA125 and HE4, respectively.

### Cytogenetic analysis

The established cell line was subjected for routine karyotype and cytogenetic analysis. Briefly, cell cultures were treated with 100 μl of KarioMAX colcemid solution (10 μg/ml; Life Techinologyes, Carlsbad, CA, USA) and incubated for 5 h (37 °C, 5% CO_2_). Cells were detached with trypsin/EDTA (0.25%), transferred to a 15 ml conical tube and centrifuged (200 g/10 min/room temperature). The cells were subjected to hypotonic treatment with 0.075 M KC1 for 8 min at 37 °C, centrifuged and fixed at least 3 times for 10 min with a freshly prepared 3:1 mixture of methanol and acetic acid. Chromosome preparations were then made by dripping the cell suspension onto cold and wet clean slides and air-drying. The chromosome analysis was performed after staining of the slides for Giemsa G-banding. Twenty good quality metaphases were analyzed in order to describe the main findings, and digital images were obtained by using the BANDview System (Applied Spectral Imaging; Carlsbad, USA).

### Cell line authentication

Analysis of short tandem repeats (STRs) was performed by Genomic Engenharia Molecular Ltda. (São Paulo, Brazil). STR analyses included markers for 21 loci on different chromosomes: D10S1237, D13S317, D16S539, D18S51, D19S433, D1S1656, D21S11, D22S1045, D2S1338, D3S1358, D5S818, D7S820, D8S1179, HUMCSF1PO, HUMFIBRA_FGA, HUMTH01, HUMTPOX, HUMVWA, Penta D, Penta E. STR profile of CAISMOV24 cell line was compared to that of other human cell lines, by using the tool available on line at http://www.dsmz.de/services/services-human-and-animal-cell-lines/online-str-analysis.html. Cell identity is expressed as an evaluation value (EV) and calculated as EV = (Number of coincident peaks of STR profiles between cell lines A and B) X 2 / Total number of peaks of STR profiles in A and B. EV values greater than 0.9 indicate that the two cell types are derived from the same origin [20].

### Comparative genomic hybridization assay

The Affymetrix Cytoscan HD platform microarray (Affymetrix, Santa Clara, USA) was employed to compare DNA alterations between the CAISMOV24 cells and their primary malignant cells. This array comparative genome hybridization platform contains 2.6 million markers for CNVs and 750,000 for single nucleotide polymorphisms (SNPs), enabling the high resolution detection of aberrations across the genome. Prior to DNA extraction, primary malignant cells were isolated from ascites by positive selection of the leukocytes, which was achieved by employing anti-CD45 MAb conjugated with beads and magnetic columns (MidiMacs separation system, Miltenyi, Bergisch Gladbach, Germany). Additionally, the cell suspension of the CAISMOV24 line was obtained by the detachment of cells from the established culture. DNA was obtained with an extraction kit based on columns with a silica membrane for DNA purification. (Illustra genomicPrep MiniSpin Kit, GE Healthcare, Buckinghamshire, UK). Finally, 250 ng of genomic DNA from CAISMOV24 cells and their primary malignant cells was used for microarray hybridization using the Cytoscan HD platform (Affymetrix) according to the manufacturer’s instructions. The Affymetrix CEL data files were converted into CYCHP files and the microarray data were analyzed using the Affymetrix Chromosome Analysis Suite 3.0 (ChAS 3.1) (Affymetrix). Analysis was performed using a setting of 50 consecutive probes and 100 kbp in length for the detection of gains or losses. The intersection of genes present in genomic alteration regions, common between the primary malignant cells and CAISMOV24 cells, was analyzed using InteractVenn software (http://www.interactivenn.net/). Relevant genes contained in the altered genomic sequences were compared with those reported by the Catalogue of Somatic Mutations in Cancer (COSMIC Welcome Trust Sanger Institute, http://cancer.sanger.ac.uk/).

### Mutation screening

DNA and RNA were obtained from cryopreserved CAISMOV24 cells at 83rd in vitro passage with extraction kits based on columns with silica membrane and designed for DNA or RNA purification respectively (Illustra genomicPrep MiniSpin Kit, GE Healthcare, Buckinghamshire, UK). DNA and RNA were quantified with the Qubit dsDNA HS Assay Kit (ThermoFisher Scinteific, Waltham, USA) and RNA BR Assay Kit (ThermoFisher Scinteific) respectively. The quality of DNA extraction products was assessed through electrophoresis in agarose (1%) stained with SYBR safe DNA Gel Stain (ThermoFisher Scinteific). RNA fragment length was assessed by a Bioanalyzer trace (DV_200_ metric) using an RNA 6000 chip on an Agilent Bioanalyzer (Agilent Technologies Inc., Santa Clara, USA). DNA products were assessed for possible mutations in *KRAS* (exon 2), *BRAF* (exon 2) and *TP53* (exon 2–11) genes by Sanger sequencing with the BigDye Terminator v3.1 Cycle Sequencing Kit (ThermoFisher Scientific) and capillary electrophoresis in an Applied Biosystems automated sequencer. Primers used for PCR are provided in Additional file 1: Table S1, and were based on the previously described by Arcila et al. [21]. Additionally, TruSight RNA Pan-Cancer Panel (Illumina, Inc., USA) was employed for transcriptome analysis of 1385 genes and 21,043 exons regions implicated in hotspot cancer pathways, following the manufacturer’s protocol. Briefly, targeted RNA-seq libraries were prepared using 50 ng of total RNA. The sample was subjected to RNA sequencing on an Illumina MiSeq (Illumina, Inc.) at 8 samples per flow cell (~3 M reads per sample). Read mapping, gene expression information, variant calling, and fusion detection were performed using the RNA-Seq Alignment App with STAR aligner on BaseSpace Sequence Hub.

## Results

### CAISMOV24 cell line establishment and in vitro growth kinetics

Primary culture with cells from ascites was mainly composed of epithelial cells, and a small number of fibroblasts. However, the number of fibroblasts decreased until disappearing along with the initial in vitro passages. As previously mentioned, the first 9 to 12 initial subcultures were performed without a regular period of time (among 3 to 4 weeks), the period in which cell proliferation was slow and unable to cover the entire culture flask surface. After this period, cell proliferation became quicker and in vitro passages for the maintenance of cell culture became regular (every 2 weeks). To evaluate the reproducibility of the cell culture transformation from primary cells into the cell line, this procedure was repeated using cells from ascites which were maintained and cryopreserved. As a result, the same pattern of development was observed.

**Fig. 2:**
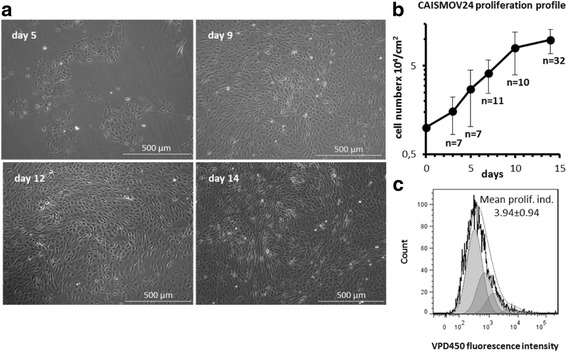
**a** Different time points of the in vitro growth of the CAISMOV24 cell line. CAISMOV24 cells were launched at 10^4^ cells/cm^2^ in HAM F10 medium supplemented with 2 mM L-glutamine and 10% fetal bovine serum. **b** Representative growth curve for the CAISMOV24 cell line, assessed from the 63rd to the 100th in vitro passages. **c** Proliferation profile of CAISMOV24 cells assessed by flow cytometry on day 5, following cell labeling with violet proliferation dye 450 (VPD450); the shaded areas represent each of the new cell generations, which retained approximately half of the VPD450 fluorescence intensity of its parent cell. Mean proliferation index of CAISMOV24 cells resulted in 3.94 ± 0.94 times

CAISMOV24 has been in continuous culture for more than 30 months and more than 100 in vitro passages. Figure 2a shows the typical epithelioid morphology of the established cell line. After cell plating, CAISMOV24 cells require 2–3 days to exhibit their fully proliferation capability, when their average doubling population time was calculated to be 71.2 h. Although the growth rate of the cell culture diminished from the 10th day, the cells continue to proliferate until covering the entire surface of the culture flask, reaching approximately 100,000 cells/cm^2^ with 96% viability (Fig. 2b). VPD450-stained CAISMOV24 cells assessed by flow cytometry allowed the mean proliferation index to be calculated as 3.94 ± 0.94 times (Fig. 2c).

### Immunophenotyping and biomarker production in culture

**Fig. 3:**
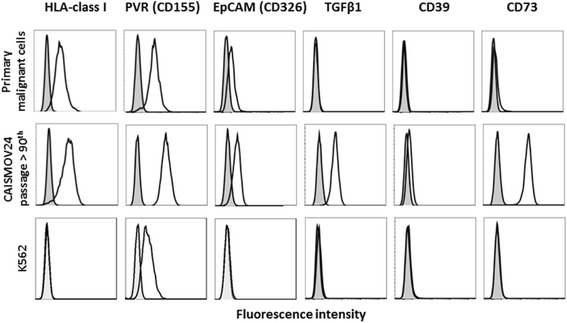
Flow cytometric profiles representative of the fluorescence intensity of the molecules, HLA-class I, PVR (CD155), EpCAM (CD326), TGF-β1, CD39 and CD73, comparing the expression of these surface molecules between the CAISMOV24 cell line and its primary malignant cells. The cells were incubated with appropriate concentrations of fluorochrome-conjugated monoclonal antibodies. After incubation, cells were washed with PBS and the final cell pellets suspended for acquisition, using a FACS Verse flow cytometer. The K562 cell line was employed as a negative control for most of the surface molecules assessed in this assay

Figure 3 compares the expression of the HLA-class I, PVR (CD155), EpCAM (CD326), TGFβ1, CD39 and CD73 surface molecules between CAISMOV24 cells and their primary malignant cells from ascites. The CAISMOV24 cell line was characterized by the expression of HLA-class I, EpCAM and PVR molecules. Additionally, CAISMOV24 cells express high levels of TGFβ1 and CD73, and low levels of CD39, molecules which are absent or modestly expressed on primary malignant cells. Serum biomarkers such as CA125 and more recently HE4 are important components in the treatment of women with adnexal masses. The production of serum biomarkers by CAISMOV24 cells in culture was confirmed by the detection of CA125 (135.80 U/ml ±56.26) and HE4 (811.45 pmol/ml ±53.53) in the supernatant of CAISMOV24 cell cultures.

### Cytogenetic analysis

**Fig. 4:**
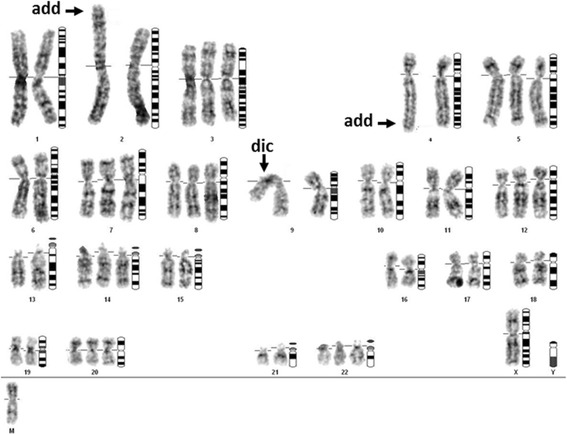
Representative G-banded karyotype of a CAISMOV24 cell with 54 chromosomes. Dic = dicentric chromosome, add = additional material of unknown origin

The chromosomes of the cell line were initially analyzed by a routine karyotyping approach in the 45th in vitro passage. Figure 4 shows one representative G-banded karyotype depicting the main findings. According to standard nomenclature, the karyotype of this cell is denoted as 53~55,XX, +1, add(1) (p36), add(2) (p25), +3, add(4) (q35), +5, +7, +8, −9, +dic(9;?) (p13;?), +12, +14, +16, −19, +20, +22, −X, +mar[cp51].

### Cell line authentication

Tables 1 and 2 summarize the main findings regarding STR analysis. Comparisons of STR profiles between CAISMOV24 and other human cell lines did not match EV values greater than 0.9, confirming the uniqueness of CAISMOV24 cell line.

**Table 1:** Short tandem repeat (STR)  analysis of CAISMOV24

Locus	Alleles
D10S1237	21,19
D13S317	12,11
D16S539	12,12
D18S51	19,12
D19S433	14,13
D1S1656	16,15.3
D21S11	32.2,30
D22S1045	16,16
D2S1338	17,17
D3S1358	17,16
D5S818	13,12
D7S820	12,8
D8S1179	13,10
HUMCSF1PO	12,12
HUMF13B	10,9
HUMFIBRA_FGA	25,23
HUMTH01	6,6
HUMTPOX	8,8
HUMVWA	17,17
Penta D	9,9
Penta E	13,7

**Table 2:** Short tandem repeat (STR) comparisons with DSMZ database

EV	Cell No. Scored
1.00–0.95	0
0.95–0.90	0
0.90–0.85	0
0.85–0.80	1
0.80–0.75	3
0.75–0.70	3
0.70–0.65	33
0.65–0.60	74
0.60–0.55	214
0.55–0.50	328
0.50–0.45	38
0.45–0.40	503
0.40–0.35	675
0.35–0.30	608
0.30–0.25	458
0.25–0.20	223
0.20–0.15	90
0.15–0.10	13
0.10–0.05	0
0.05–0.00	10

STR profile of CAISMOV24 cell line was compared to that of other 3274 human cell lines available at DSMZ site, http://www.dsmz.de/services/services-human-and-animal-cell-lines/online-str-analysis.html; Number of cell lines scored among different evaluation values (EV)

### Comparative genomic hybridization assay

**Fig. 5:**
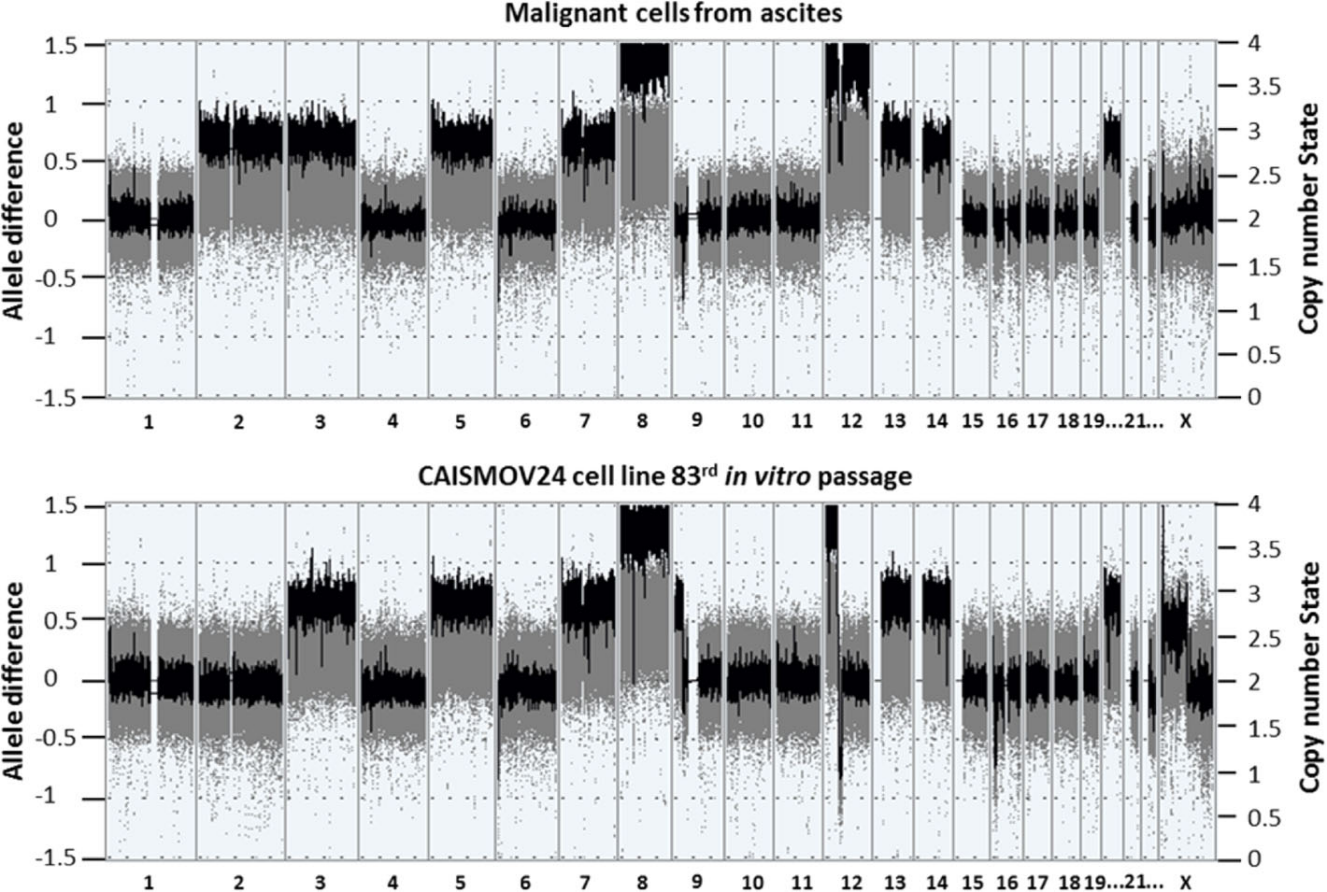
Image captured from software ChAS (Affymetrix, USA) summarizing chromosomal aberrations found across the genome of the CAISMOV24 cell line compared with its primary malignant cells. Extensive alterations, involving long genomic sequences, were detected in chromosomes 3, 5, 7, 8, 12, 13, 14, 19 and X, both in the CAISMOV24 cell line and its primary malignant cell. X axis = Chromosomes; Left y axis = Allele differences (gray); Right y axis = Copy number state (black)

CAISMOV24 cell line and its primary malignant cells were compared for chromosomal aberrations. For this purpose, a comparative genomic hybridization assay was employed. Table 3 summarizes the main findings regarding CNVs. The total number of CNVs increased with the number of in vitro passages, with 28 detected in the primary malignant cells and 37 in the cell line assessed at the 83rd in vitro passage. Chromosomes 4, 10, 15, 17, 18 and 21 remained unaltered, both in the primary malignant cells and in the cell line. Additionally, in eight of the altered chromosomes, the number of CNVs remained the same for the primary malignant cells and the cell line. The most affected chromosomes (with three or more CNVs) in the primary malignant cells were chromosomes 7, 8, 12, 20 and X, which represented 60.7% (17 in 28) of the total CNVs. Chromosomes 1, 7, 8, 9, 12, 16 and 20 from the CAISMOV24 cell line had high numbers of CNVs (three or more CNVs), which represented 67.6% (25 in 37) of the total CNVs. Although CNVs were mostly represented by gains of genomic sequences (86% of CNVs in primary malignant cells and 78% in CAISMOV24 cell line), CNVs also included losses of genomic sequences and hemizygous deletions of duplicated chromosome regions, characterizing copy neutral loss of heterozygosity (cnLOH, genotyping provided in Additional file 2: Table S2, Additional file 3: Table S3 and Additional file 4: Table S4). Among losses of genomic sequences, there was one detected on chromosome 9 (ch9p21.3) of the primary malignant cells that was not detected in CAISMOV24 cell line. Extensive alterations, involving long genomic sequences, were detected in chromosomes 2, 3, 5, 7, 8, 12, 13, 14, 19 and X (Fig. 5). The total size of genomic sequences contained in CNVs was higher in CAISMOV24 cells (1,971,963 kbp) than in the primary malignant cells (1,394,479 kbp).

**Table 3:** Comparison of copy number variations (CNVs) between CAISMOV24 cell line and its primary low-grade serous ovarian carcinoma cells

Primary maligant cells						CAISMOV24					
Chromosome	CNV	Type	Size (kbp)	Cytoband interval	Gene Count	Chromosome	CNV	Type	Size (kbp)	Cytoband interval	Gene Count
1	1	Gain	221	1p36.32(3,331,773–3,552,456)×3	6	1	3	Gain	113,168 144,332 317,481	1p36.33(2,173,472–2,286,640)×0–1 1p36.33(900,361–1,044,693)×2–3 1p36.32(3,324,208–3,641,689)×0–1	3 8 7
2	1	Gain	242, 771	2p25.3q37.3(12,770–242,783,384)×28–29	1494	2	1	cnLOH	242,76	2p25.3q37.3(15,702–242,775,910) hmz	1494
3	1	Gain	197,79	3p26.3q29(61,891–197,851,986)×3	1279	3	2	Loss Gain	276 197,79	3q12.1(98,596,503–98,872,626)×0–1 3p26.3q29(61,891–197,851,986)×3	1 1278
5	1	Gain	180,606	5p15.33q35.3(113,576–180,719,789)×3	1035	5	1	Gain	180,606	5p15.33q35.3(113,576–180,719,789)×3	1035
6	1	Loss	127	6p25.3(254,282–381,137)×1	1	6	1	Loss	127	6p25.3(254,253–381,137)×1	1
7	3	Gain Gain Gain	7818 66,570 83,982	7q11.21q11.23(66,698,378–74,516,616)×3 7p22.3q11.21(43,360–66,613,020)×3 7q11.23q36.3(75,137,713–159,119,707)×3	58 418 659	7	4	Gain Gain Gain Gain	1695 3221 72,589 80,786	7q11.23(72,692,112–74,386,749)×3 7q11.23q21.11(75,034,632–78,255,196)×3 7p22.3q11.23(43,360–72,632,831)×3 7q21.11q36.3(78,333,779–159,119,707)×3	33 39 436 626
8	4	Loss Gain Gain Gain	140 4398 39,089 99,432	8p11.22(39,247,097–39,386,952)×2 8p11.22p11.1(39,388,765–43,786,723)×4 8p23.3p11.22(158,048–39,246,760)×4 8q11.1q24.3(46,863,521–146,295,771)×4		8	4	Loss Gain Gain Gain	140 4398 39,089 99,432	8p11.22(39,247,097–39,386,952)×1 8p11.22p11.1(39,388,765–43,786,723)×4 8p23.3p11.22(158,048–39,246,760)×4 8q11.1q24.3(46,863,521–146,295,771)×4	2 33 292 511
9	1	Loss	209	9p21.3(21,887,365–22,096,124)×1	4	9	3	Gain Gain Gain	679 3244 21,879	9q34.3(139,282,807–139,961,930)×3 9p21.3(22,086,839–25,330,810)×291–292 9p24.3p21.3(203,861–22,082,884)×2–3	45 5 102
						11	1	Gain	364	11p15.5(241,986–606,294)×3	21
12	4	Gain Gain Gain Gain	9834 11,031 28,314 84,394	12q12q13.12(39,535,139–49,369,195)×29–30 12p13.33p13.2(173,786–11,204,306)×39–40 12p13.2q12(11,207,579–39,521,153)×39–40 12q13.12q24.33(49,385,726–133,777,902)×39–40	68 192 149 778	12	3	Loss Gain Gain	9499 11,276 23,369	12q12q13.11(39,441,095–48,940,506)×1 12p13.33p13.2(173,786–11,450,109)×39–40 12p13.2p11.1(11,466,434–34,835,837)×39–40	51 198 139
13	1	Gain	95,671	13q11q34(19,436,286–115,107,733)×3	459	13	1	Gain	95,671	13q11q34(19,436,286–115,107,733)×3	459
14	1	Gain	86,774	14q11.2q32.33(20,511,672–107,285,437)×2–3	770	14	2	Gain Gain	13,876 71,619	14q32.12q32.33(93,408,967–107,285,437)×29–30 14q11.2q32.12(20,511,672–92,130,966)×29–30	238 521
16	1	cnLOH	90,074	16p13.3q24.3(89,560–90,163,275) hmz	955	16	5	Loss Gain Gain Loss cnLOH	124 143 236 291 82,965	16p13.3(7,094,531–7,218,941)×1 16p13.3(1,986,979–2,129,528)×3 16p13.3(1,052,879–1,288,518)×3 16p13.3(7,441,425–7,732,737)×1 16p13.3q24.3(7,198,476–90,163,275) hmz	1 18 6 1 734
19	1	Gain	1142	19p13.3(260,911–1,403,381)×24–25	51	19	1	Gain	1256	19p13.3(260,911–1,517,292)×24–25	58
20	3	Gain Gain Gain	1499 5355 55,959	20p13(61,568–1,560,550)×3 20q13.32q13.33(57,560,776–62,915,555)×3 20q13.32q13.33(57,560,776–62,915,555)×3	34 106 515	20	3	Gain Gain Gain	557 14,149 47,64	20p12.1(14,319,185–14,875,738)×3 20p13p12.1(61,568–14,210,343)×3 20p12.1q13.33(15,275,354–62,915,555)×3	2 134 518
22	1	Gain	512	22q11.22(22,943,460–23,455,803)×25–26	6	22	1	Gain	458	22q11.22(22,997,802–23,455,803)×24–25	4
X	3	Gain Gain Gain	182 201 384	Xq21.31(88,604,293–88,786,664)×3 Xp22.33(2,956,273–3,156,774)×3 Xq21.2(85,297,084–85,680,929)×3	0 1 2	X	1	Gain	74,778	Xp22.33q13.3(168,546–74,946,70n)×2–3	494

*CNV* copy number variation; Column “Gene Count” refers to number of genes found in CNV sequence, *cnLOH* Copy neutral loss of heterozygosity; Cytoband interval based on assembly human genome hg19

The chromosomal regions affected by CNVs included 10,523 genes. Of these, 8798 genes were common for CNVs found either in the CAISMOV24 cell line or its primary malignant cell, 710 genes were present only in CNVs of the CAISMOV24 line, whilst 1015 genes were present only in the CNVs of the primary malignant cell (InteractVenn http://www.interactivenn.net/).

### Mutation screening

Screening for typical mutations of serous ovarian carcinomas found that CAISMOV24 harbored KRAS mutation, together with wild type TP53, characterizing the cell line as low-grade serous ovarian carcinoma. Furthermore, transcriptome analysis confirmed the typical KRAS mutation (rs121913529; HGVS nomenclatures: NM_004985.4:c.35G > T, NP_004976.2:p.Gly12Val) frequently observed in low-grade serous ovarian carcinomas. Transcriptome analysis also showed absence of gene fusions, and revealed several genes with SNP, in which its clinical relevance for the development of the serous ovarian carcinoma is unknown (Additional file 5: Table S5). Table 4 shows 15 variant genes without SNP location record and their frequency of alterations in CAISMOV24 cell line was higher than 0.35.

**Table 4:** Gene mutations without SNP location record and with high alteration frequencies in CAISMOV24 cell line

Gene	Chr	Position	Depth	Ref	Alt	Alt Freq	Variant Type
AKAP9	chr7	91,652,181	18	C	CAAC	0.39	inframe insertion
HIST1H1D, HIST1H2APS3	chr6	26,235,122	186	G	A	0.39	missense variant, downstream gene variant
AKAP12	chr6	151,674,121	585	A	AGGA	0.41	inframe insertion
SPEN	chr1	16,202,753	20	G	A	0.45	missense variant
NCOR2	chr12	124,812,039	117	G	T	0.47	missense variant
EP400	chr12	132,554,157	32	G	A	0.59	missense variant
ZNF687	chr1	151,259,035	5	G	C	0.60	missense variant, upstream gene variant
NOTCH1	chr9	139,393,579	38	C	T	0.63	missense variant
HMGA1, C6orf1	chr6	34,211,292	59	A	AA	0.78	frameshift variant, feature elongation, downstream gene variant
WHSC1L1	chr8	38,148,080	20	C	T	0.85	missense variant
KIF5B	chr10	32,311,775	7	C	G	1.00	splice donor variant
PPP2R1B	chr11	111,631,542	2	C	T	1.00	splice donor variant
RELN	chr7	103,230,181	2	T	C	1.00	missense variant
AKAP9	chr7	91,652,181	18	C	CAAC	0.39	inframe insertion
HIST1H1D, HIST1H2APS3	chr6	26,235,122	186	G	A	0.39	missense variant, downstream gene variant

*Chr* chromosome, *Ref* reference nucleic acid, *Alt* altered nucleic acid, *Alt Freq* frequency alteration

## Discussion

In this study a new low-grade serous ovarian carcinoma cell line, named as CAISMOV24, was characterized in terms of its in vitro cell growth, production of soluble biomarkers and expression of cell surface molecules. Additionally, CAISMOV24 was molecularly characterized and compared to its primary malignant cells for genomic alterations. In vitro models of well-characterized low-grade serous ovarian cell lines are currently limited in the literature. CAISMOV24 resulted from the in vitro spontaneous immortalization of primary malignant cells from ascites that was associated with a low-grade serous ovarian carcinoma. Although malignant cells of ovarian neoplasia from either tumor tissue or ascites can be cultivated in vitro for a limited period, only a minority of the primary cell cultures may become cell lines [14]. Spontaneous immortalization of primary cultures of malignant cells is an event occurring at a very low frequency. As an example, O’Donnell et al. [11] reported the occurrence of only one spontaneous immortalization among 156 primary cultures generated from epithelial ovarian neoplasia, while all the other cultures entered senescence between the 2nd and 8th in vitro passages. The spontaneous immortalization of primary cultures is frequently assigned to the genetic instability of malignant cells and accumulation of genomic alterations, such as the gain or loss of genomic sequences, during in vitro culturing. However, this is a poorly understood and little documented phenomenon. Additionally, these genomic alterations can affect cell lines, driving them away from the cognate tumor profile and, thus, limiting their usefulness as experimental models.

Genomic alterations in low-grade serous ovarian carcinomas have been described as intermediate between borderline and high-grade serous ovarian carcinomas [22–24]. However, CNVs can be remarkably heterogeneous either in number or in their genomic location, among different low-grade tumors [25]. Although our results showed that CAISMOV24 cell line had arisen alongside the accumulation of genomic alterations, most of them were related to CNVs already present in the primary malignant cells. Additionally, some of these CNVs were reported to be shared by low-grade serous ovarian carcinomas. Thus, among the genomic alterations detected in the CAISMOV24 cell line and its primary malignant cells, copy number gains in chromosomes 8, 12 and 20 were mentioned by Fernandez et al. [25] as commonly detected in low-grade serous carcinoma cell lines. Similarly, chromosome 4 was among those that did not harbor genomic alterations.

Our results showed that few CNVs were found exclusively in the cell line or in its primary malignant cells. Particularly, hemizygous deletion at ch9p21.3 was observed predominantly in primary malignant cells, but not in CAISMOV24 cells. This observation supported the low-grade origin of our primary cells, since hemizygous or homozygous deletions at ch9p21.3 have been reported as one of the most frequent copy number loss observed in low-grade serous ovarian carcinomas [22, 25]. However, the absence of this CNV in CAISMOV24 cells suggests that cell line could have emerged from specific cell clones of the primary malignant cells. Similarly, Lee et al. [26] showed that peritoneal metastasis arises from cellular clones with little accumulation of CNV in relation to its primary tumor in patients with high-grade serous ovarian cancer. Taken together, these observations suggest that similar mechanisms could be operating in vivo and in vitro systems. Thus, CAISMOV24 cell line shall have evolved from a process involving both, expansion of specific cell clones in addition to the enlargement or loss of altered pre-existing genomic sequences in primary malignant cells.

Although copy number alterations and gene mutations are caused by independent processes, it has been suggested that CNV profiles can be used for a better characterization among different histotypes of epithelial ovarian cancer [12], since they could harbor histotype-specific gene mutations. Additionally, CNVs could encode genes which drive growth [13, 27] and could alter gene expression or disrupt regulatory mechanisms leading to phenotypic alterations of the malignant cells in culture. Typically, low-grade serous ovarian carcinoma has few somatic mutations, which are mainly found in genes associated with the mitogen-activated protein kinase pathway such as *KRAS*, *BRAF* and *NRAS* [23, 25, 28]. *KRAS* and *BRAF* mutations are found in a proportion of low-grade serous carcinomas and, frequently, they are mutually exclusive. This is in contrast to high-grade serous carcinomas, which mutations include *TP53* gene and rarely *KRAS* [25, 29]. Accordingly, our results showed that CAISMOV24 cell line harbors *KRAS* mutation, which is relatively frequent in recurrent low-grade serous carcinomas [30]. The transcriptome analyses not only confirmed CAISMOV24 cell line as a low-grade serous carcinoma, but also revealed several genes with SNPs, which clinical relevance for development of serous ovarian carcinomas has being under evaluation. In this context, *HMGA1* over-expression was observed in epithelial ovarian carcinomas [31], while *NOTCH1* has been correlated with ovarian cancer development and a poor prognosis [32, 33].

In this study, immunophenotyping of the CAISMOV24 cell line was performed in an attempt to characterize the cells in relation to the expression of surface molecules, frequently observed in epithelial ovarian carcinomas, which have also been implicated in the failure of an immune response against tumors. Thus, the epithelial origin of CAISMOV24 cells was confirmed by the expression of EpCAM and PVR, two molecules expressed by ovarian epithelial cells, which are frequently overexpressed in ovarian carcinomas [19, 34, 35]. Additionally, expression of the HLA-class I molecule was not lost as a consequence of the malignant transformation of the ovarian epithelial cells [36, 37]. Our results showed that CAISMOV24 cells express high levels of TGF-β1 and CD73, and low levels of CD39, molecules that were absent or modestly expressed on the primary malignant cells. All of these molecules have been implicated in impaired immune response against ovarian carcinomas, either by direct TGF-β1-mediated effects [9, 38, 39], or by indirect effects resulting from the breakdown of ATP into adenosine by CD73, which promotes the immunosuppression of T cytotoxic and NK lymphocytes [9, 37]. Serum biomarkers such as CA125 and more recently HE4 are important components in the treatment of women with adnexal masses. HE4 is a glycoprotein with low expression in normal ovarian tissue, higher in non-ovarian cancer tissue and highest in ovarian cancer tissue. HE4 as a single tumor marker has been reported to be as good as CA125 for the detection of ovarian cancer. HE4 and CA125 together enhance the sensitivity and specificity for the diagnosis of ovarian cancer [7, 8]. In this study, the in vitro production of CA125 and HE4 by CAISMOV24 cells was confirmed, stressing the similarities between the cell line and its primary malignant cells.

## Conclusion

In this study, the CAISMOV24 cell line was compared to its primary malignant cells. Our results corroborate with the idea that genomic alterations, depicted by CNVs, can be used for subtyping epithelial ovarian carcinomas. Additionally, CAISMOV24 cell line was characterized as a low-grade serous ovarian carcinoma, which still resembles its primary malignant cells.

## Additional files

Additional file 1: Table S1. Primers. Primers used for PCR amplification prior to Sanger sequencing of *KRAS*, *BRAF* and *TP53* genes. (DOCX 15 kb)

Additional file 2: Table S2. LOH ch2p25.3 CAISMOV24. Genotyping of hemizygous deletions of duplicated chromosome regions (copy neutral loss of heterozygosity or cnLOH) in chromosome 2 of CAISMOV24 cell line. (XLSX 5208 kb)

Additional file 3: Table S3. LOH ch16p13.3 primary cells. Genotyping of hemizygous deletions of duplicated chromosome regions (copy neutral loss of heterozygosity or cnLOH) in chromosome 16 of primary malignant cells. (XLSX 1738 kb)

Additional file 4: Table S4. LOH ch16p13.3 CAISMOV24. Genotyping of hemizygous deletions of duplicated chromosome regions (copy neutral loss of heterozygosity or cnLOH) in chromosome 16 of CAISMOV24 cell line. (XLSX 1750 kb)

Additional file 5:Table S5. CAISMOV24 PanCancer transcriptome. Transcriptome data of CAISMOV24 cell line. (XLSX 531 kb)

## Abbreviations

ATP: Adenosine triphosphate
CA125: Cancer antigen 125
CCLE: Cancer Cell Line Encyclopedia
CD: Cluster of differentiation
cnLOH: Copy neutral loss of heterozygosity
CNV: Copy number variation
COSMIC: Catalogue of Somatic Mutations in Cancer
DNAM-1: DNAX accessory molecule-1
EDTA: Ethylenediamine tetraacetic acid
EOC: Epithelial ovarian cancer
EpCAM: Epithelial cell adhesion molecule
FACS: Fluorescence-activated cell sorting
FBS: Fetal bovine serum
FSC: Forward scatter
HE4: Human epididymis protein 4
Mab: Monoclonal antibody
PVR: Poliovirus receptor
SNP: Single nucleotide polymorphism
SSC: Side scatter
VPD450: Violet proliferation dye 450
