# Supplementary material for: CAISMOV24, a new human low-grade serous ovarian carcinoma cell line
Source: BMC Cancer. 2017 Nov 13;17:756. doi: 10.1186/s12885-017-3716-4 (PMC5683553; doi:10.1186/s12885-017-3716-4)
Supplement: Supplementary file 1 — Primers. Primers used for PCR amplification prior to Sanger sequencing of KRAS, BRAF and TP53 genes. (DOCX 15 kb) [file 12885_2017_3716_MOESM1_ESM.docx]

Table S1. Primers used for PCR amplification prior to Sanger sequencing of *KRAS* exon 2, *BRAF* exon 15 and *TP53* exons 2 to 11.

| **Primer** | **Sequence** |
| --- | --- |
| *KRAS exon 2*F | 5-GTGTGACATGTTCTAATATAGTCA-3 |
| *KRAS* exon 2R | 5-CTGTATCAAAGAATGGTCCTGCAC-3 |
| *BRAF exon* 15F | 5-TCATAATGCTTGCTCTGATAGG-3 |
| *BRAF* exon 15R | 5-GGCCAAAAATTTAATCAGTGG-3 |
| *TP53* exon 2+3F | 5’-gtaaaacgacggccagtCACTGGCATGGTGTTGGGGGAG -3’ |
| *TP53* exon 2+3R | 5’-taatacgactcactatagggTGTAGATGGGTGAAAAGAGCAGTCA-3’ |
| *TP53* exon 4F | 5’-gtaaaacgacggccagtGGACTGACTTTCTGCTCTTGTCTTT -3’ |
| *TP53* exon 4R | 5’-taatacgactcactatagggCAGAGATCACACATTAAGTGGGTAA -3’ |
| *TP53* exon 5F | 5’-gtaaaacgacggccagtCTCTCTAGCTCGCTAGTGGGT-3’ |
| *TP53* exon 5R | 5’-taatacgactcactatagggCGAAAAGTGTTTCTGTCATCCAAAT -3’ |
| *TP53* exon 6F | 5’-gtaaaacgacggccagtGCCATGGCCATCTACAAGCA -3’ |
| *TP53* exon 6R | 5’-taatacgactcactatagggTGGGGTTATAGGGAGGTCAAA-3’ |
| *TP53* exon 7F | 5’-gtaaaacgacggccagtACAGGTCTCCCCAAGG-3’ |
| *TP53* exon 7R | 5’-taatacgactcactatagggAAACTGAGTGGGAGCAGTAAGGAGA-3’ |
| *TP53* exon 8+9F | 5’-gtaaaacgacggccagtGGACAAGGGTGGTTGGGAGTAGA-3’ |
| *TP53* exon 8+9R | 5’-taatacgactcactatagggCCCAATTGCAGGTAAAACAGTCAAG-3’ |
| *TP53* exon 10F | 5’-gtaaaacgacggccagtCAGTTTCTACTAAATGCATGTTGCT -3’ |
| *TP53* exon 10R | 5’-taatacgactcactatagggATACACTGAGGCAAGAATGTGGTTA-3’ |
| *TP53* exon 11F | 5’-gtaaaacgacggccagtCATCTTGATTTGAATTCCCGTTGT-3’ |
| *TP53* exon 11R | 5’-taatacgactcactatagggCACCAGTGCAGGCCAACTTGTTCAG -3’ |

*Obs1*. Small caps letters correspond to the sequenced of the M13 universal (5’-gtaaaacgacggccagt-3’) and T7 (5’-taatacgactcactataggg-3’) primers, incorporated in the PCR primers to allow sequencing of all amplicons with the same primers.

*Obs2*. Primers for KRAS and BRAF are those described by Arcila et al. [21].
